# Copper–Ruthenium Composite as Perspective Material for Bioelectrodes: Laser-Assisted Synthesis, Biocompatibility Study, and an Impedance-Based Cellular Biosensor as Proof of Concept

**DOI:** 10.3390/bios12070527

**Published:** 2022-07-14

**Authors:** Daniil D. Stupin, Anna A. Abelit, Andrey S. Mereshchenko, Maxim S. Panov, Mikhail N. Ryazantsev

**Affiliations:** 1 Nanotechnology Research and Education Centre RAS, Saint Petersburg Academic University, 8/3 Khlopina Street, 194021 St. Petersburg, Russia; anna.abelit@gmail.com; 2 Institute of Chemistry, Saint Petersburg State University, 7/9 Universitetskaya nab., 199034 St. Petersburg, Russia; a.mereshchenko@spbu.ru (A.S.M.); m.s.panov@spbu.ru (M.S.P.); 3 Center for Biophysical Studies, St. Petersburg State Chemical Pharmaceutical University, Professor Popov Str., 14, Lit. A, 197022 St. Petersburg, Russia

**Keywords:** copper biocompatibility, impedance spectroscopy, biocompatible materials, biocompatible electrodes, biosensors

## Abstract

Copper is an inexpensive material that has found wide application in electronics due to its remarkable electric properties. However, the high toxicity of both copper and copper oxide imposes restrictions on the application of this metal as a material for bioelectronics. One way to increase the biocompatibility of pure copper while keeping its remarkable properties is to use copper-based composites. In the present study, we explored a new copper–ruthenium composite as a potential biocompatible material for bioelectrodes. Sample electrodes were obtained by subsequent laser deposition of copper and ruthenium on glass plates from a solution containing salts of these metals. The fabricated Cu–Ru electrodes exhibit high effective area and their impedance properties can be described by simple R-CPE equivalent circuits that make them perspective for sensing applications. Finally, we designed a simple impedance cell-based biosensor using this material that allows us to distinguish between dead and alive HeLa cells.

## 1. Introduction

Nowadays, bioelectronics is actively involved in many areas of bioscience and biotechnology, such as biosensing [1,2,3,4,5,6,7,8,9,10,11,12,13,14], vision, hear, bone prosthetic care [15,16,17,18,19,20,21,22,23,24,25,26,27], artificial cardiac pacemakers development [28,29], medical diagnostics [30,31,32,33], neuroscience [34,35,36], etc. Such great achievements became possible both due to the emergence of well-developed semiconductor microelectronics technologies in the second half of the 20th century [37,38,39,40], and due to the intensive development of biotechnologies, e.g., cytotechnologies, in the 20th and 21st centuries [41,42,43,44]. The further progress in this field will in large part depend on the development of new bioelectrodes that serve as an interface for transferring information between living matter and semiconductor microelectronics and for the electrical stimulation of cells and tissues.

At present, precious metals or their alloys are the most common materials used for fabrication of bioelectrodes (see ref. [2] p. 70 and refs. [45,46,47]). Despite the remarkable electrochemical properties and biocompatibility of the noble metals, there are also disadvantages such as their expensiveness and difficulties in processing [40]. Copper is an inexpensive material that is widely used in electrical engineering due to its remarkable electrical properties. Copper electrodes are often used to manufacture electrochemical sensing devices [48,49,50,51] (e.g., glucose sensors) and as a base for skin electrodes [52,53,54]. However, due to the high toxicity of copper and copper oxide [55,56,57], copper electrodes can not replace expensive noble metal electrodes in many important fields of bioelectronics such as impedance-based in vitro and in vivo cell and tissue studies (for example, using the ECIS method [6]), neuronal electrophysiological investigation with multi-electrode arrays [34], etc.

As it was demonstrated in several recent studies, the modification with biocompatible substances can significantly improve biocompatibility of copper. Among the modified Cu-based materials with good biocompatibility are Ti–Cu–Zr alloys [58], steel–copper composites [59], Ti–Cu alloys [60], Fe–Cu compounds [61], copper with polymerized coating [62], and Cu–Al–Zn–Ni–Mn alloys [63]. Following this line of thought, we applied the technique of laser-induced metal deposition from solution [64] to synthesize bimetallic electrode materials containing copper and such a biocompatible metal as ruthenium [65]. A pure Cu electrode was also synthesized as a control sample. The obtained Cu-based electrodes are promising candidates for sensing applications, because they exhibit high effective area and can describe impedance properties by a simple R-CPE equivalent circuit. In addition, the Cu–Ru electrode has proved to be highly biocompatible in contrast to pure the Cu electrode. Further, the Cu–Ru electrode was used to design a simple impedance-based biosensor allowing us to distinguish between dead and alive HeLa cells.

## 2. Materials and Methods

### 2.1. Synthesis of Cu-Based Electrodes

All chemicals used in the current work were analytically graded and were purchased from Sigma Aldrich (St. Louis, MO, USA). The solution compositions utilized for synthesis of the electrode materials are shown in Table 1.

Laser-assisted fabrication of Cu microstructures (electrodes) and their further modification with Ru was performed using a simple and cheap approach that was successfully applied for similar purposes in our previous works. This approach is based on the method of laser-induced metal deposition from solution (LCLD) [64,66,67,68,69,70]. In general, LCLD deals with the reactions of metal reduction and its subsequent deposition on the surface of different dielectrics (glass, glass-ceramics, etc.) that occurs in a local volume of a solution within the focus of the laser beam.

**Figure 1:**
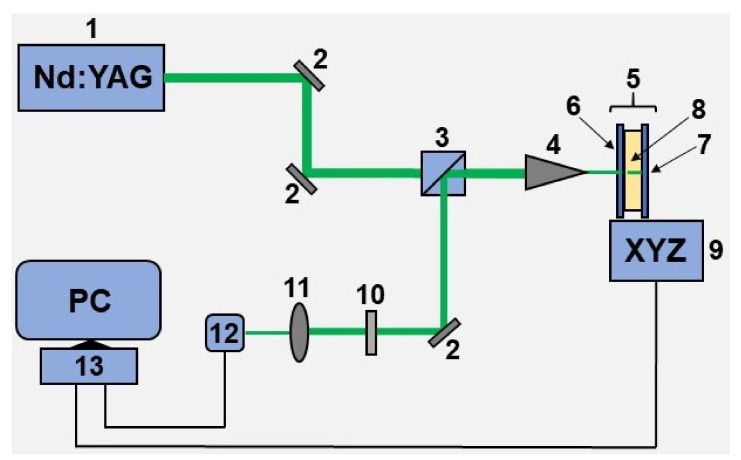
The schematic picture of the experimental setup used for the fabrication of Cu-based electrodes. 1—Nd:YAG 532 nm blue cw (continuous-wave) laser, 2—Al mirror, 3—splitting cube, 4—microscope objective, 5—experimental chemical cell, 6—glass window, 7—electrode glass substrate, 8—chemical solution (see Table 1), 9—XYZ motorized stage, 10—attenuation filter, 11—focusing lens, 12—web-camera, 13—personal computer.

The detailed description of the experimental setup used for the fabrication of Cu-based electrodes can be found elsewhere [64]. Briefly, the 532 nm output from a diode-pumped continuous-wave solid-state Nd:YAG laser (Changchun, China) was used as a light source (Figure 1). After passing through two Al mirrors and an optical separation cube, the laser output was focused on (5 µm laser spot) the experimental cell, i.e., between a solution and a dielectric substrate (glass) using a standard microscope objective (15 mm focus length). In order to produce electrodes of various geometry, this cell can be translated into three dimensions using a computer-controlled XYZ-motorized stage. In turn, the 532 nm light was partially reflected back by the cell surface toward an optical separation cube with subsequent redirection to another Al mirror. Further, the redirected beam light traveled to a web camera that was used for monitoring and regulating the metal deposition process. In order to avoid optical damage to the web camera, the intensity of the reflected laser output was attenuated by a neutral density filter (fractional transmittance 25%).

In this study, we performed a two-stage synthesis of Cu-based electrodes on the surface of the glass. In the first stage, we fabricated copper microstructures (electrodes) of 10 mm length and ∼150 µm width at a laser power density of ∼51 mW/µm^2^ and at a scanning speed of 5 µm/s. In the second stage, the presynthesized Cu structures were modified by laser deposition of Ru on their surface at the same scanning speed and at laser power density of 66 mW/µm^2^. The difference in laser power densities for the first and second stages of synthesis was caused by the different initiation thresholds of the reduction reactions of copper and ruthenium.

### 2.2. SEM and XRD Diagnostics

The morphology of Cu-based electrodes was studied using a Zeiss Supra 40 VP scanning electron microscope (Oberkochen, Germany) coupled with an INCA X-Act EDX analyzer (Oxford Instruments, UK) for characterization of their atomic composition. The X-ray diffraction analysis (XRD) of Cu-based electrodes was carried out on a Bruker D2 Phaser diffractometer equipped with a LynxEye detector (Karlsruhe, Germany) using Cu $K_{\alpha }$ (1.542 Å) radiation in the 2θ angle range of 0–100°.

### 2.3. Admittance Measurement

The admittance spectra were obtained using the Fourier immittance spectroscopy method (FFT-EIS) [4,71,72]. The measurements were performed on a homemade setup discussed in ref. [73]. The AKIP 3413/3 generator (AKIP, Russia) was used as an excitation voltage source switched in the sine-sweep regime. These experiments were conducted in the frequency range between 2 Hz and 40 kHz with a 2 Hz resolution and at excitation voltage with a 15 mV amplitude. The glass filled with PBS solution (phosphate-buffered saline, Biolot, Russia) was used as an electrochemical cell, in which the investigated electrode and reference platinum electrode with a large area were embedded. EIS measurements were started after the disappearance of the faradaic current.

**Figure 2:**
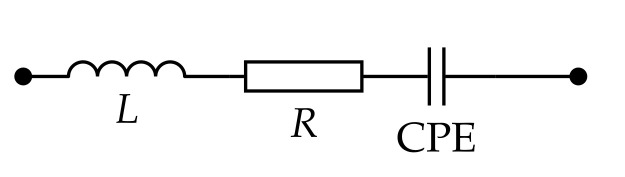
The equivalent scheme for describing Cu-based electrodes. Here, *L*∼10 mH is parasitic inductance caused by finite-time response of the ammeter.

The admittance spectra were analyzed using complex non-linear least squares (CNLS) method [74] in the NELM package for MatLab [73] (can be obtained by request). The scheme presented in Figure 2 was used for CNLS spectra analysis. Here, CPE is constant phase element [75] with impedance equal to

(1) $$Z=\frac{1}{W{\left(i\omega \right)}^{\alpha }},$$

where α is non-ideality parameter, which is also called the *constant phase element exponent*, and *W* is pseudo-capacitance with dimension of $S\cdot s^{\alpha }$.

For taking into account the delay between excitation voltage and current response measurements by ADC [76], the parameter Δt was introduced into the model as follows:

(2) $$Y_{m}=Y_{s}\times e^{i\omega \Delta t},$$

where $Y_{m}$ is a model, which was used for CNLS approximation, $Y_{s}$ is admittance of the scheme Figure 2, and ω is an angular frequency. The measurements were repeated 5 times to calculate 99% confidence intervals.

### 2.4. Biocompatibility Study

We used the following experimental scheme to study the biocompatibility of the fabricated Cu-based electrodes. On the day before the experiment, the HeLa cells [43] obtained from the Bank of Cell Cultures of the Institute of Cytology of the Russian Academy of Sciences were seeded on the surface of these electrodes. Then, they were incubated for 24 h in a CO_2_ incubator at 37 °C and 5% CO_2_ in DMEM medium (Sigma-Aldrich, MO, USA), containing 5% fetal bovine serum and 0.1% gentamicin.

Further, each electrode was washed three times with PBS and treated first with DiBAC dye (visualization of the membrane, Thermo Fisher Scientific, MA, USA) and then with propidium iodide dye (PI, visualization of dead cell nuclei, Thermo Fisher Scientific, MA, USA). Each dye was diluted by 1000 times with PBS. All electrodes were treated with each diluted dye for 1 min. Next, they were placed into a Petri dish with PBS and examined using a Leica 4000 DM fluorescence microscope (Leica Microsystems GmbH, Germany). Consequently, we have used the “I3” cube to obtain DiBAC fluorescence, and the “N21” cube to obtain propidium iodide fluorescence.

The recorded microscopic photographs were further processed using the MatLab package to create combined pseudo-color images. The monochrome, green, and red channels correspond to the image in the transfer-light, the DiBAC, and PI fluorescence, respectively.

### 2.5. Cell Adhesion Study

To study the adhesion of the HeLa cells to Cu-based electrodes, we examined them on a Zeiss Observer.Z1 confocal microscope (Carl Zeiss Microscopy GmbH, Germany) [77] using the following protocol. The day before the experiment, the cells were seeded on the electrode surface and incubated under the same conditions as described in the subsection “Biocompatibility study”. Then, the samples were washed three times with PBS and stained with PI diluted by 1000 times. After this procedure, the cells were stained with the Deep Red Cell Mask dye (membrane visualization, Thermofisher Scientific, USA) and fixed with 3.7% paraformaldehyde. Finally, the cells were treated by Fluoroshield with DAPI dye (cell nuclei visualization, Sigma Aldrich, St Louis, MO, USA) and covered with a coverslip that was later glued to the samples by nail polish. In order to obtain high-resolution images of the studied samples, immersion oil was placed between the 100× objective and the coverslip. For analysis, we selected the cells that did not exhibit PI fluorescence and, therefore, were alive before paraformaldehyde treatment.

## 3. Results

Cu and Cu–Ru electrodes were synthesized using the experimental setup (Figure 1) and methodology described in the section “Materials and methods”. In the context of biosensing applications in general and applications such as bioelectrodes for impedance-based spectroscopy in particular, the following physical and biological properties of an electrode are desirable: for higher sensitivity of analytical measurement electrode surface has to be developed, the admittance spectra of the electrodes should allow fitting by the simple equivalent scheme, the electrode must be biocompatible, and adhesion of a cell on the electrode surface has to be as good as possible. Investigation of all these properties both for Cu and Cu–Ru electrodes are given below in this section.

### 3.1. Surface Characterization and Elemental Composition

**Figure 3:**
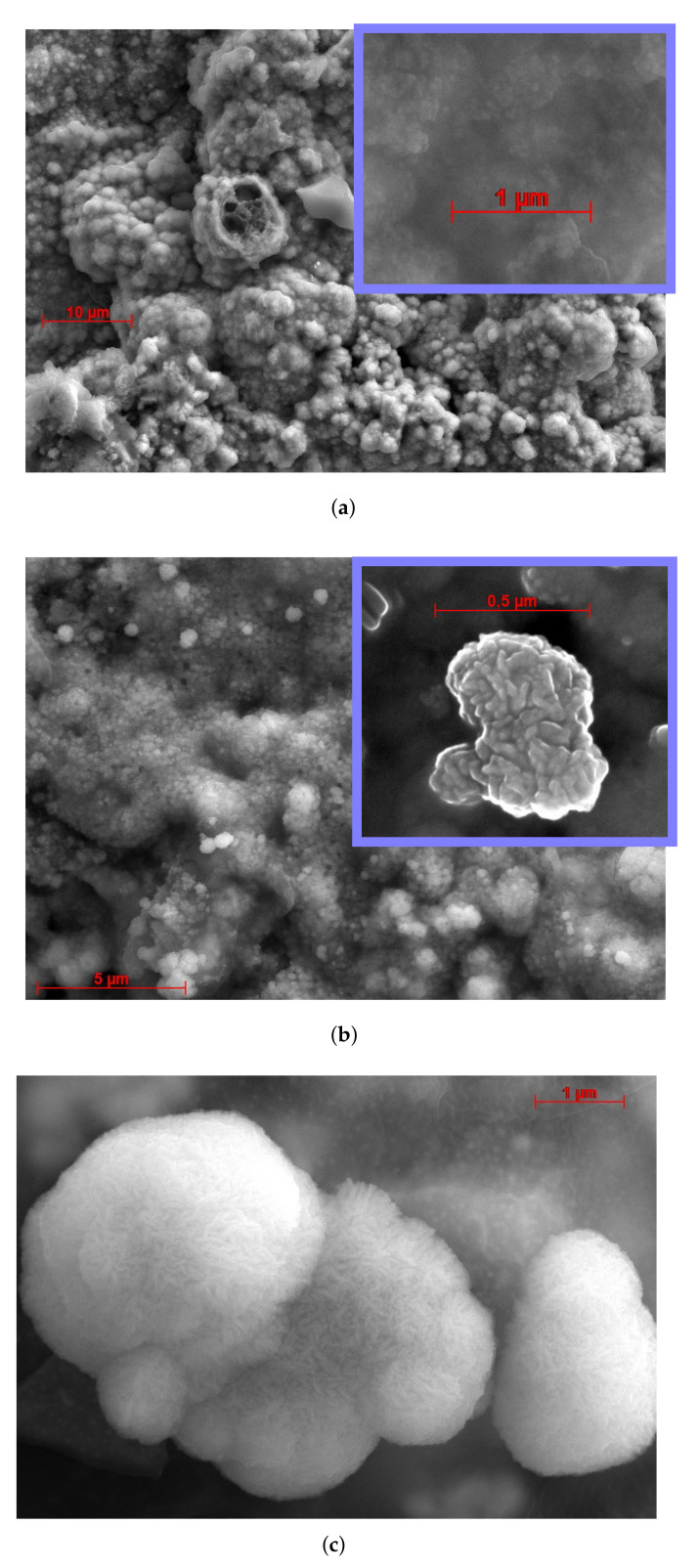
SEM images of Cu-based electrodes. (**a**) Pure Cu electrode; (**b**,**c**) Cu–Ru electrode. The inserts in blue squares show the microstructure of these electrodes. SEM images were obtained using the secondary electrons mode with 20 kV accelerating voltage. One can see that both electrodes have developed porous surface and demonstrate an obvious hierarchical structure—large pores and protrusions that contain small irregularities. Moreover, the surface of Cu–Ru electrode consists of ruthenium islands spread on top of the Cu layer (see Figure 4b).

The results of the SEM studies are presented in Figure 3. Both electrodes have porous and developed morphology. Their surfaces exhibit hierarchic structure, i.e., they contain large pores composed of smaller irregularities. The modification of Cu electrode with Ru does not qualitatively change their hierarchical structure. However, this process naturally reduces the pore size because of the growth of Ru particles inside the Cu pores. Such a hierarchical organization effectively increases the surface of the electrodes without compromising their linear dimensions. It is important for biological applications, including the determination of the cell impedance and measurement of neuroactivity. The obtained results agreed well with the impedance measurements discussed in Section 3.2. From manual pore size measurements (Figure 3), we found that the average pore sizes for Cu and Cu–Ru electrodes are 1.4(1) and 0.50(7) µm, respectively. Analogically, we measured the pore density *D* from Figure 3. It was found that *D* equals 50/100 and 300/100 µm^−2^ for Cu and Cu–Ru electrodes, respectively. Using these values, we were able to estimate the increase in effective area with respect to the area of the planar electrodes as 97% for Cu and 90% for Cu–Ru (see Appendix B).

**Figure 4:**
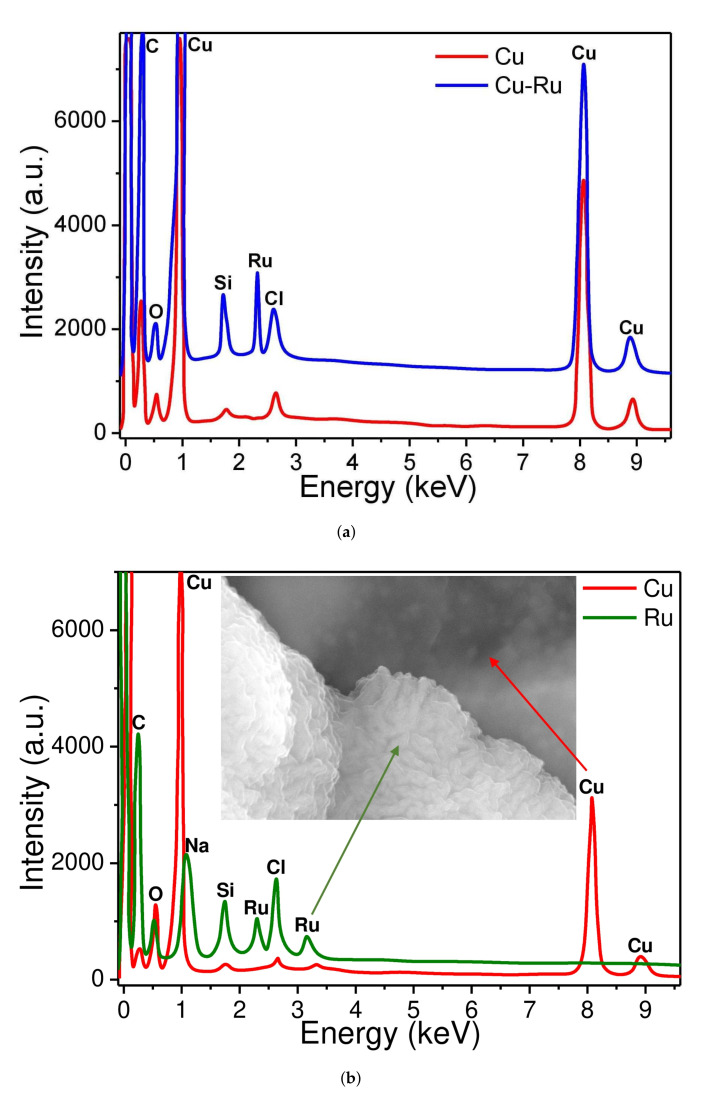
The results of X-ray microanalysis confirm the the elemental composition of Cu (**a**) and Cu–Ru (**b**) electrodes. One can see that Cu–Ru electrode consists of ruthenium islands located on top of copper (here, insert image is magnified SEM photograph Figure 3c, scale bar corresponds to 200 nm). The C, Na, Cl, O, and Si peaks correspond to the elements of the substrate material (glass).

**Figure 5:**
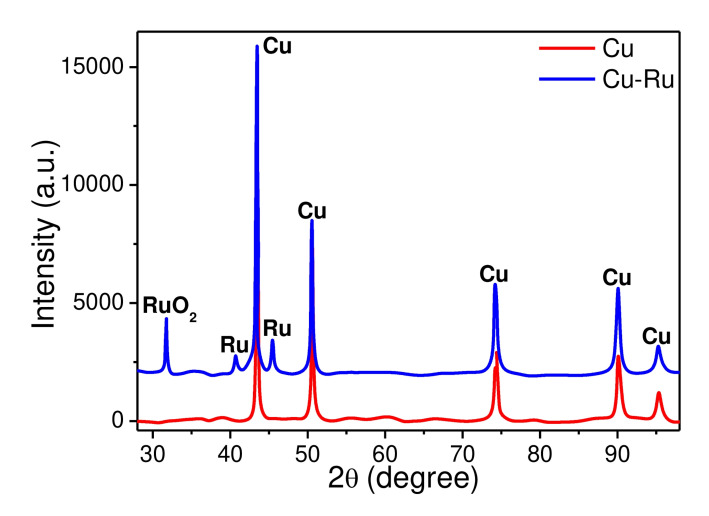
The XRD patterns of Cu and Cu–Ru electrodes fabricated at the optimized physical (laser power, scanning speed) and chemical parameters (composition of solutions).

The elemental composition of Cu-based electrodes and their crystalline structure investigation are presented in Figure 4. According to these data, all electrode materials are very pure and polycrystalline. The surface of the Cu–Ru electrode deserves special attention. It consists of ruthenium islands, which are located on the pure copper layer (see Figure 3c and Figure 4b). It should be noted that these islands have zigzag nanopores resembling those seen in Ru-based electrode fabricated in our previous work [64]. The contents of copper and ruthenium observed in the Cu–Ru electrode according to EDX (Figure 4) were 71% and 12%, respectively. The EDX analysis also showed the presence of oxygen, sodium, chlorine, silicon, and carbon in the sample, which can be attributed to the substrate material (glass). The X-ray phase analysis (XRD) was performed to determine the phase composition of the obtained Cu-based electrode materials. The obtained data showed the presence of a polyphase multicomponent system containing metallic and oxide phases (Figure 5). For both samples, the metallic phase is copper, whereas ruthenium is an additional metal phase in the Cu–Ru electrode. In addition, the low-intensity reflexes of RuO_2_ were observed for Cu–Ru. It should be mentioned that the results of the XRD analysis of Cu-based electrodes are consistent with EDX data.

### 3.2. Admittance Study

**Figure 6:**
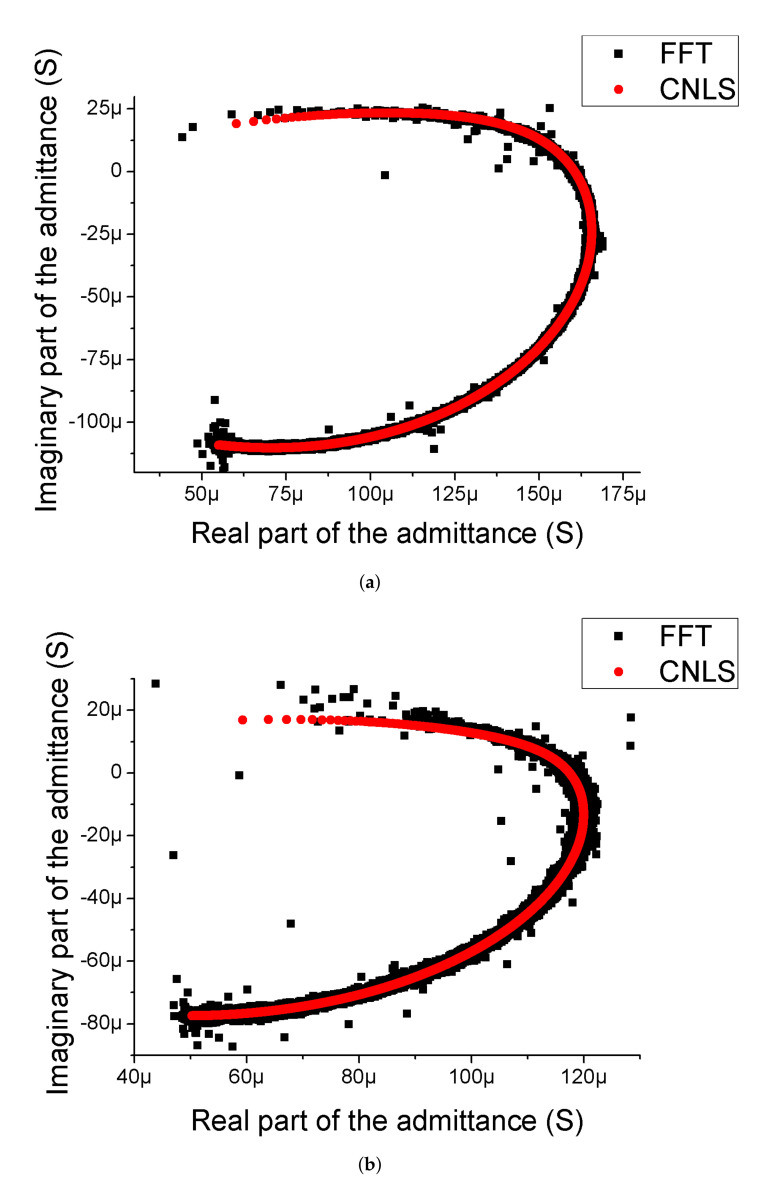
The admittance loci and their CNLS-approximation for Cu (**a**) and Cu–Ru (**b**) electrodes. The black squares correspond to the experimentally obtained admittance spectra using the FFT-EIS, and the red circles denote the CNLS fitting of the experimental data with an equivalent scheme shown in Figure 2. As one can see, the admittance spectra of all electrodes have an arc shape indicating that the surfaces of these electrodes are developed and porous. The difference in the shape of the spectra is associated with different values of α caused by different pore sizes and the structure of the studied electrodes (see Figure 3 and Table 2).

The obtained admittance spectra and their CNLS approximations are presented in Figure 6. As can be concluded from these data, all spectra are arc-shaped (not semicircle) indicating that the surface of the electrodes is porous and developed [4,71,78] (compare with Figure 3). Moreover, these spectra can be perfectly fitted by the equivalent scheme (Figure 2) that contains only one R-CPE branch. Thus, the surface morphology of the electrodes for the most part should not reveal areas with significantly different types of irregularities (e.g., areas with nanopores separated by areas with micropores), which is also confirmed by SEM in Figure 3. Analyzing the data presented in Table 2, we can observe that the exponents α for Cu-based electrodes are lower than 1/2. At first glance, this observation seems disturbing, since typical capacitance dispersion exponent lies in the range of 1/2≤α≤1 [79,80,81,82]. In addition, the value α=1/2 is the limiting value in the case of the infinite pore with pure capacitance surface/electrolyte impedance [78]. Therefore, for the α value lower than one half, we must assume that surface/electrolyte impedance of the pores in the electrodes should *a priori* demonstrate capacitance dispersion effect confirming high porosity of Cu-based electrodes. The more detailed explanation of this observation is presented in Appendix A. We also observed that α-values for Cu–Ru electrodes are usually higher than α-value for Cu electrode. This may be due to the clogging of the pores of the copper layer caused by ruthenium deposition during the second stage of synthesis of the Cu–Ru electrode (see Section 2.1). This process decreases the copper pore size leading to a decrease in the effective surface area (see Section 3.1) and an increase in the α-value as a result of deviation from the infinite pore limiting case (see Appendix A).

Interestingly enough, the Ru-based electrode obtained in our previous work [64] had large pores with small zigzag nanopores and “plane” regions containing zigzag nano-sized irregularities. The existence of these two types of pores resulted in the appearance of two arcs in the admittance spectra. In opposite, the Cu–Ru electrode described in this work has no “plane” regions and, hence, its admittance locus has only one arc. So it is clear that the use of copper reduces the equivalent circuit of Ru-based bioelectrodes simplifying the analysis of the bioimpedance data.

### 3.3. Biocompatibility and Cells Adhesion

**Figure 7:**
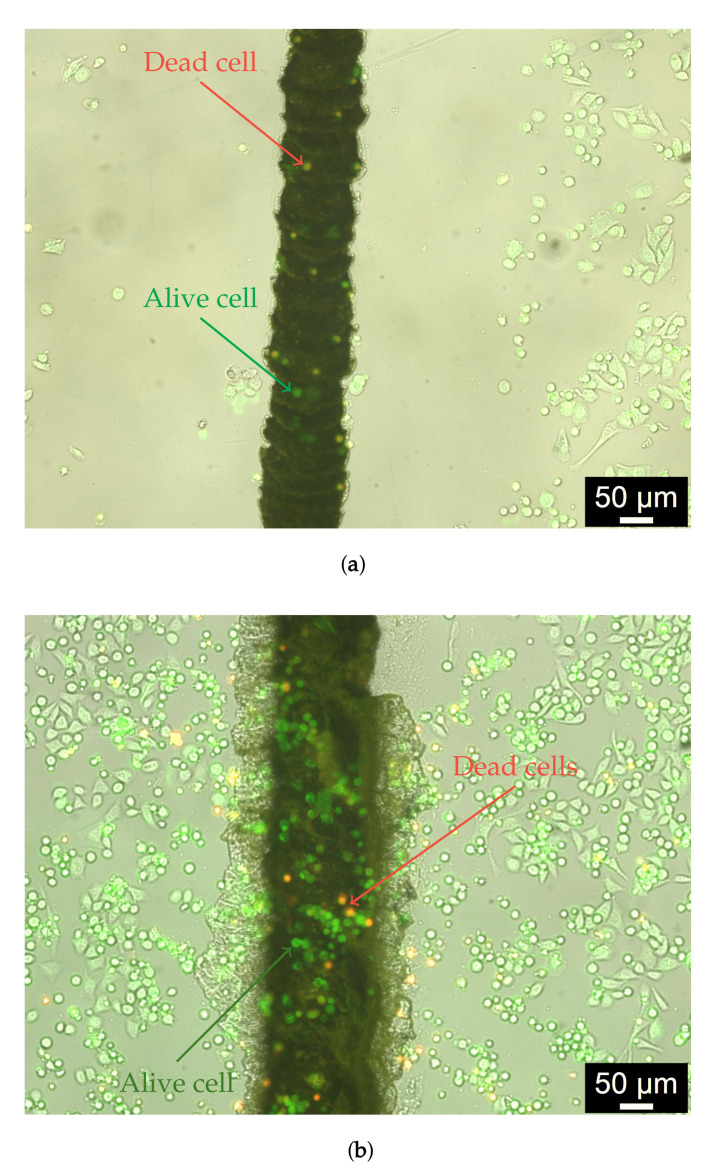
Investigation of biocompatibility of Cu (**a**) and Cu–Ru (**b**) electrodes. The dead and alive cells are shown in yellow and green, respectively (the examples of the dead and alive cells are shown by arrows). Cu–Ru electrode exhibits much better biocompatibility in comparison with pure Cu electrode (90 vs. 30% alive cells).

The results of the biocompatibility studies of Cu-based electrodes are shown in Figure 7 (the green spots in a pseudo-color photograph indicate alive cells after incubation for 24 h). The Cu electrode has much lower biocompatibility compared to the Cu–Ru electrode, and only 30 ± 10% of the cells seeded on the Cu surface survived after one day of incubation. In turn, the surface of the Cu–Ru electrode reveals 91 ± 4% of alive cells. The higher biocompatibility of Cu–Ru electrodes can be explained by the presence of biocompatible ruthenium [65] islands on the surface of Cu–Ru electrode (see Figure 3b,c and Figure 4b). The high reproducibility of biocompatible properties of the copper–ruthenium material was confirmed by a low value of the relative error.

**Figure 8:**
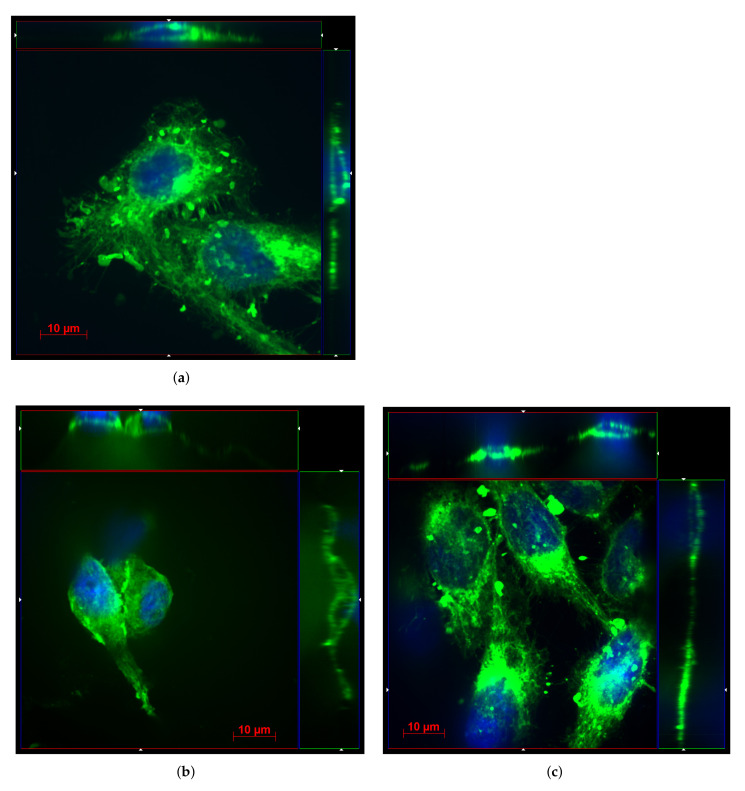
Confocal tomography images of the HeLa cells seeded on top of glass (**a**), Cu (**b**), and Cu–Ru (**c**) electrodes. Here, the red lines are referred to *x* axis, the green lines correspond to the *z* axis, and the blue lines refer to the *y* axis. The ends of cut-lines are marked by white triangles. One can see that the HeLa cells on top of Cu-based electrodes (**b**,**c**) have a fusiform shape similar to those observed on glass (**a**). Moreover, they duplicate the morphology of the electrodes, meaning that Cu-based electrodes are biocompatible.

The quality of the cell adhesion on the surface of Cu-based electrodes is presented in cut-view tomographic images (Figure 8). These pictures contain the top-view (located between red (*x* axis) and blue lines (*y* axis)) and the cross-section views of the HeLa cells [located between green (*z* axis) and blue (*y* axis) lines as well as between red and green lines]. The ends of cutting lines, across which the images in cross-section were depicted, are marked by white triangles. All images are presented in pseudocolor: the green channel corresponds to membrane visualization using Deep Red Cell Mask dye and the blue channel corresponds to nuclei visualization using DAPI dye. The natural cell adhesion on glass is presented for comparison in Figure 8a.

The obtained data show that the cell adhesion on the surface of Cu-based electrodes is ideal: (1) all cells have a natural fusiform shape; (2) all cells duplicate the shape of the electrode surface in the *z*-direction; (3) all cells create healthy focal contacts. In addition, despite the fact that the height of the electrode surface can vary within a few micrometers, the cells can grow on top of the electrodes in a monolayer manner. Thus, the Cu-based electrodes synthesized in this work are very promising for in vitro cell investigation using ECIS [6] methodology as well as for neuronal cell studies [34].

### 3.4. Biosensor as a Proof of Concept

**Figure 9:**
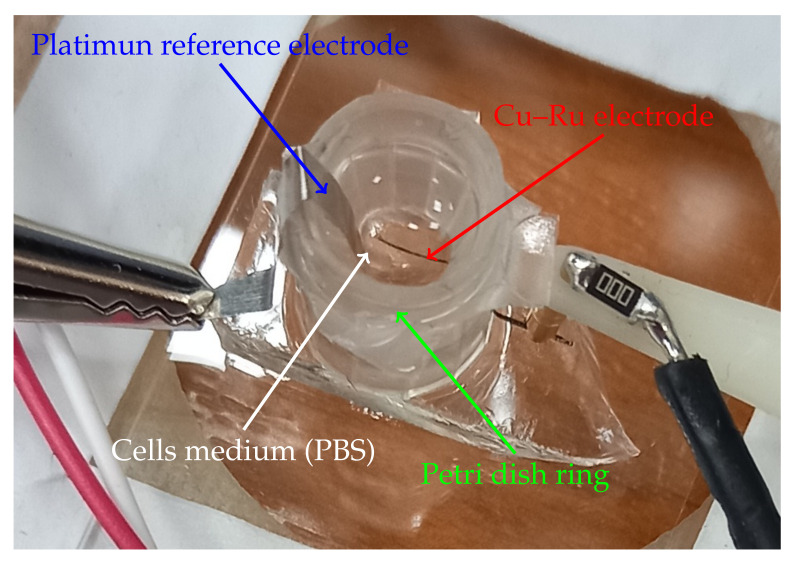
Macrophotograph of the copper–ruthenium biosensor.

For the design of an impedance-based biosensor (Figure 9), the Cu–Ru electrode with the best biocompatibility was chosen. Then, it was glued to a plastic Petri dish ring (1.1 cm in height and 8 mm in diameter) using PDMS (polydimietisiloxane). One day before the experiment, we seeded HeLa cells on top of the Cu–Ru electrode and incubated them in DMEM medium (Sigma Aldrich, St Louis, MO USA) with 5% fetal bovine serum and 0.1% gentamicin. Directly before the experiment, we changed the DMEM medium in the Petri dish with phosphate-buffered saline (PBS) containing propidium iodide dye (PI). A platinum plate was used as a reference electrode. Further, we have started impedance measurements of the Cu–Ru electrode with HeLa cells. After 3.5 min, the PI-PBS medium was replaced with PI-PBS solution containing an excess of the Triton X-100 detergent (1:1000 *v*/*v*). The impedance was measured as cell index at the frequency of 35 kHz

(3) $$\mathrm{Cell}\;\mathrm{Index}=\frac{Z(t)-Z(0)}{Z(0)},$$

where Z(0) is impedance magnitude of Cu–Ru electrode at the beginning of the experiment, and Z(t) is impedance magnitude of Cu–Ru electrode at time *t*. The impedance was measured using adaptive-filtering impedance spectroscopy [73].

**Figure 10:**
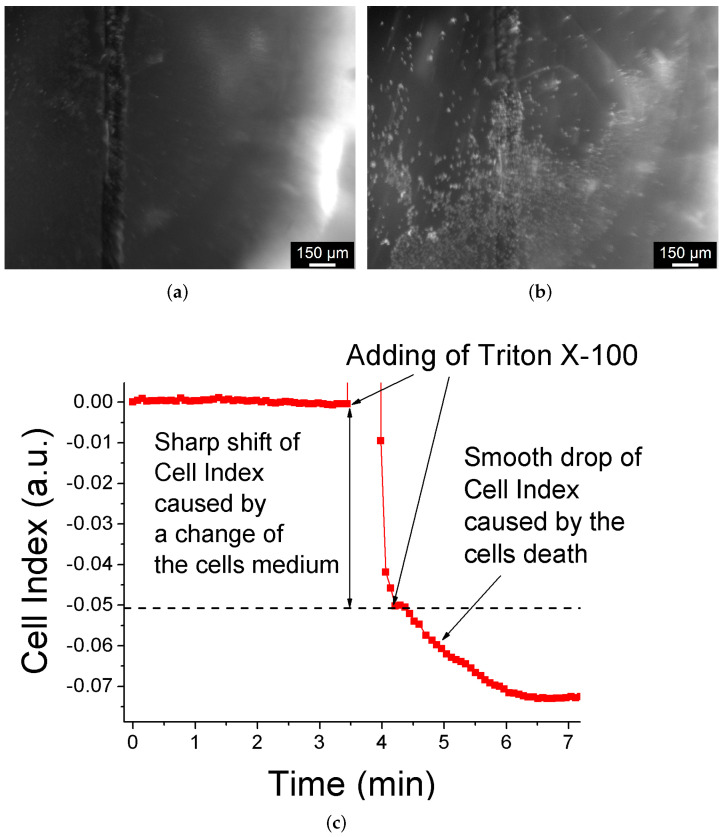
Estimation of the viability of the cells on the surface of the Cu–Ru electrode. Panels (**a**,**b**) correspond to fluorescence photographs (“N21” cube) of Cu–Ru electrode before and after Triton X-100 addition, respectively. It is clear that the cells were alive before the Triton X-100 addition since there is no fluorescence of propidium iodide. On the other hand, the cells became dead after Triton X-100 addition, which is confirmed by PI fluorescence (bright white dots). Panel (**c**) corresponds to the time evolution of the cell index. The obtained data indicate that before detergent was added when the majority of the cells on the electrode were alive, the cell index was stable at zero value. After adding detergent, almost all cells became dead leading to a slow and smooth decrease of the cell index. The sharp rise and drop of the cell index during Triton X-100 addition (∼3.5–4.0 min) can be attributed to manipulations with the cells medium (i.e., replacing and adding).

The results of biosensing application of Cu–Ru electrode are presented in Figure 10. One can see that before Triton X-100 addition the cells were alive (Figure 10a) and their cell index (Equation (3)) was stable in time for 3.5 min (Figure 10c). On the other hand, after Triton X-100 addition, the cells became dead (Figure 10b), and their cell index started rapidly decreasing. This behavior can be explained by damage to the cells membrane caused by Triton X-100 detergent leading to an increase in the current flow through cells-substrate [4,5,14]. Thus, the obtained results confirm the efficiency of Cu–Ru electrodes with respect to biosensing applications.

## 4. Conclusions

Herein we reported our investigations that aim to search for inexpensive biocompatible electrode materials with properties optimal for biosensing applications. We obtained two types of electrodes (Cu and Cu–Ru) and studied their structure, biocompatibility, adhesion, and impedance properties. The Cu–Ru composite was fabricated using laser modification of the presynthesized copper structures by ruthenium. It was shown that such a modification significantly increases the biocompatibility and adhesion ability of pure Cu. Indeed, the majority of the HeLa cells seeded on the surface of the Cu–Ru electrode survived in contrast to the Cu electrode. The high biocompatibility can be attributed to the presence of biocompatible ruthenium islands distributed along the surface of the copper layer. In addition, the electrical properties of the fabricated Cu-based electrodes are found to be very promising for cell-based biosensor devices. As a proof-of-concept application, we proposed a simple impedance-based biosensor that allows us to distinguish between dead and alive HeLa cells. Future improvement of the proposed Cu–Ru biosensor can be achieved through the use of lithographic post-processing of the LCLD materials for the creation of an array of cell-sized electrodes. This multielectrode geometry will allow studying the individual cells. It will also pave the way for the development of platforms for the investigation of neurons and other excitable cells, such as photoreceptors. In addition, the combination of the proposed Cu–Ru sensors with microfluidic technologies can be used to study the cells with poor adhesion, such as erythrocytes or K562.

Finally, the overall results of this work expand the scope of the application of biocompatible materials with untypical electrochemical properties.

## Figures and Tables

**Table 1:** Solution components used for laser-induced synthesis of metallic and bimetallic electrodes based on Cu and Ru. Here, DMF is *N*,*N*-dimethylformamide.

Electrode Material	Reagent, (mM)	Solvent
Cu	copper(II) chloride dihydrate (CuCl${}_{2}\cdot$2 H${}_{2}$O), (2)	H_2_O
	potassium sodium tartrate tetrahydrate (KNaC${}_{4}$H${}_{4}$O${}_{6}\cdot$4H${}_{2}$O), (7)	
	sodium hydroxide (NaOH), (10)	
Cu–Ru	triruthenium dodecacarbonyl (Ru${}_{3}$(CO)${}_{12}$), (3)	DMF

**Table 2:** Typical results of the CNLS fitting of the admittance spectra of Cu-based electrodes. The accuracy is calculated as 99% confidence intervals.

Parameter	*R*, kΩ	*W*, *S*·*s*${}^{\alpha }$	α
Cu	4.60(9)	3.5(3) × $10^{-5}$	0.28(1)
Cu–Ru	7.5(1)	3.2(9) × $10^{-5}$	0.34(3)

**Figure A1:**
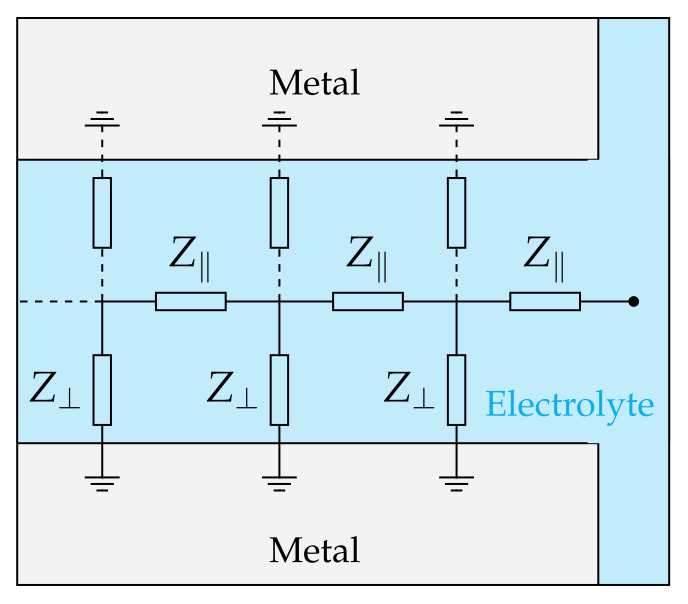
Equivalent scheme of an infinite pore according to Levie’s theory. The direction of the pore deepening is shown from right to left. Here, $Z_{⊥}$ is the impedance of the metal/electrolyte interface, and $Z_{\|}$ is the impedance of the pore cross-section.

## Data Availability

Not applicable.

## Appendix A. On the Origin of the CPE Exponent below 1/2

As it was mentioned in the section “Results”, for an α value lower than one-half, it is necessary to assume that the surface/electrolyte impedance of the pores in the electrodes should a priori demonstrate capacitance dispersion effect. Indeed, if we calculate the impedance $Z_{P}$ of the infinite pore in the Levie’s transmission-line approximation approach (Figure A1) using Feynman trick [83], for $|Z_{\|}\left|\ll \right|Z_{⊥}|$, we should get the classical Levie’s result:

(A1) $$Z_{P}=\sqrt{Z_{⊥}Z_{\|}}.$$

In ref. [78] Levie calculated the pore impedance using assumption that the flat metal/electrolyte interface has double-layer pure capacitive impedance $Z_{⊥}=1/\left(i\omega C\right)$ and the plausible assumption that the pore cross-sectional impedance is purely resistive, i.e., $Z_{\|}=R$, which leads to well-known result:

(A2) $$Z_{P}=\sqrt{\frac{R}{C}}\times \frac{1}{{\left(i\omega \right)}^{1/2}},$$

where *C* is the double layer capacitance (per unit length) and *R* is the resistance of the pore cross-section.

According to Equation (A2), the CPE exponent is equal to one-half in the limiting case of an infinite pore with pure capacitance surface/electrolyte impedance. However, at the same time, if the metal/electrolyte interface $Z_{⊥}$ *a priori* has pseudocapacitance behavior with α<1, then, according to Levie’s works [78,84], it is possible to have CPE with a value of α below 1/2 at the electrode/electrolyte interface. Indeed, if $Z_{⊥}{=1/[W\left(i\omega \right)}^{\beta }]$, then, from Equation (A1), we obtain:

(A3) $$Z_{P}=\sqrt{\frac{R}{W}}\times \frac{1}{{\left(i\omega \right)}^{\alpha }},$$

where α=β/2≤1/2. Therefore, the phenomenon of the dispersion of capacitance with a CPE exponent lower than one-half has a clear physical meaning. Such an effect can be observed either if the electrode surface contains pores consisting of smaller pores [85,86], or if it contains pores in which electrochemical reactions and diffusion processes occur. The latter explains the presence of a Warburg-type impedance [4] at the metal/electrolyte interface [84,87]. For our samples, the first assumption seems to be the most probable, since according to Figure 3, Cu and Cu–Ru electrodes have uniformly distributed pores containing smaller irregularities. Moreover, since the α values are near 1/4, in accordance to the Itagaki [86] interpretation, we can conclude that these electrodes have only a two-level hierarchy, i.e., they only consist of pores with smaller pores, and these smaller pores have a non-developed surface. Thus, the proposed LCLD electrodes are promising not only for biosensing, but also for creation of factor devices [88,89,90,91].

## Appendix B. Estimation of the Effective Surface Area

In this section, we derived the formula for the estimation of the effective electrode area. It was assumed that each pore, numbered by index *j*, has a semi-spherical shape with a diameter of $d_{j}$. Further, the surface area of the planar electrode was designated as $S_{el}=H\times W$ (here *W* is the width of the electrode and *H* is its length), whereas the number of the pores locates on $S_{el}$ was assigned as *N*. As a result, in order to estimate the electrode effective area, the value of $S_{wp}$ (the area of pores) should be subtracted from the electrode area $S_{el}$, and then the result of subtraction should be added to the total area of the semi-spherical pores $S_{p}$. Obviously,

(A4) $$S_{wp}=\frac{\pi }{4}\sum_{j=1}^{N}d_{j}^{2},$$

i.e., it is a sum of the areas of the circles with diameter $d_{j}$, and

(A5) $$S_{p}=\frac{2\pi }{4}\sum_{j=1}^{N}d_{j}^{2},$$

i.e., it is a sum of the areas of the semi-spheres with diameter $d_{j}$. Thus, the effective surface area is equal to:

(A6) $$S_{eff}=S_{el}-S_{wp}+S_{p}=S_{el}\left(1+\frac{\pi }{4}D\langle d_{j}^{2}\rangle \right),$$

where *D* is number of the pores per unit area (density of pores) and $\langle d_{j}^{2}\rangle$ is mean-square value of the pore size. Finally, the expression in parentheses in the last right-hand side of the Equation (A6) represents the coefficient of increase of the effective surface area.
